# The burden of multimorbidity in patients with TB–HIV: a retrospective cohort analysis

**DOI:** 10.5588/ijtldopen.26.0114

**Published:** 2026-10-12

**Authors:** K.J Hill, N.C Owaro, C. Sekaggya-Wiltshire, P. Mubiri, E.L.A Odongpiny, J Matovu, L Alinaitwe, F Aber, S Mpagama, S.E. Mills, H.R Stagg, D.J. Sloan

**Affiliations:** 1 Infection and Global Health Department, School of Medicine, University of St Andrews, St Andrews, UK;; 2 Infectious Diseases Institute, Makerere University, Kampala, Uganda;; 3 Kibong’oto Infectious Diseases Hospital, Sanya Juu, Tanzania;; 4 Department of Infectious Disease Epidemiology, London School of Hygiene and Tropical Medicine, London, UK.

**Keywords:** tuberculosis, Uganda, non-communicable disease, treatment outcomes

## Abstract

**BACKGROUND:**

Multimorbidity (i.e., the co-existence of two or more long-term health conditions [LTCs]) is increasing in high-TB-burden countries. Few studies have estimated the prevalence of multimorbidity in patients with TB, and none in TB–HIV. This retrospective cohort study used HIV clinic data from the Infectious Diseases Institute in Kampala, Uganda, to determine the prevalence and patterns of multimorbidity in patients with TB–HIV at the time of TB diagnosis, and its effect on TB treatment outcomes.

**METHODS:**

Adults living with HIV and diagnosed with TB between 1 January 2014 and 14 November 2024 were included. Clinical, laboratory, and prescription records were used to categorise LTCs. The prevalence and patterns of multimorbidity were examined and their effect on WHO-defined TB treatment outcomes was assessed using regression models.

**RESULTS:**

Among 993 included patients, 76.1% (95% confidence interval: 73.4%–78.8%) had multimorbidity and 34.3% (31.4%–37.4%) had three or more LTCs. Anaemia (52.1%) and undernutrition (34.3%) were most prevalent. Multimorbidity was associated with a 3.70% (−0.35%–7.74%, *P* = 0.07) absolute increase in the adjusted risk of an unsuccessful treatment outcome.

**CONCLUSION:**

Multimorbidity was highly prevalent at TB diagnosis in this cohort of people with TB–HIV, highlighting the complexity of care required alongside prescription of anti-TB therapy.

The burden of non-communicable disease (NCD) and multimorbidity – that is, the co-existence of two or more physical or mental long-term health conditions (LTCs) – is increasing in low- and middle-income countries (LMICs).^1–3^ This is driven by economic development, urbanisation, and lifestyle change.^1–3^ Uganda exemplifies this epidemiological transition, with an increasing prevalence of NCD risk factors and a rising proportion of deaths attributable to NCDs.^4,5^ However, TB and HIV remain highly prevalent.^6^ People living with HIV (PLWHIV) are at higher risk of TB disease, and co-infection increases mortality risk.^7^ These infections are co-managed through well-established protocols.^7^ Despite shared biological and social determinants driving overlap between TB and NCDs, and calls to urgently address multimorbidity in patients with TB, the management of NCDs in patients with TB lags behind.^7–10^ Epidemiological studies have largely focused on TB with single LTCs. African studies which have looked at multiple LTCs report that 25.3%–45% of patients with TB have at least two LTCs additional to TB.^11,12^ None have studied this in TB–HIV; however, over a third of patients with TB in East Africa are living with HIV.^11^

The objective of this retrospective cohort study was primarily to describe the prevalence and patterns of multimorbidity and single LTCs in PLWHIV in Kampala, Uganda, at time of TB diagnosis and secondarily to examine the effect of multimorbidity on TB treatment outcomes.

## METHODS

The Infectious Diseases Institute, Makerere University (IDI), is a tertiary HIV treatment facility located at Mulago National Referral Hospital in Kampala, Uganda. It manages the Integrated Clinic Enterprise Application (ICEA), an electronic longitudinal dataset of routinely collected health care data from over 22,000 PLWHIV from 2007 to present.^13^ Adults (≥18 years) with a documented diagnosis of TB (drug-susceptible [DS-TB] or drug-resistant) treated at IDI between 1 January 2014 and 14 November 2024 were included from the time of their TB diagnosis. For patients with multiple TB episodes during this timeframe, only the first was included. Analyses examining TB treatment outcomes were restricted to DS-TB.

### Data extraction and cleaning

Data inclusive of demographics, details of TB diagnosis, treatment and outcome, date of HIV diagnosis, laboratory results, prescription records, clinical observations, and documented medical history were extracted and cleaned for inconsistencies and outliers. TB episodes were linked to the nearest clinic visit within 30 days of diagnosis. Individuals missing both body mass index and blood pressure measurements at time of TB diagnosis – reflecting incomplete clinical records with extensive missingness in other covariates – were excluded from the cohort. Within this study, LTCs were classified from clinical, laboratory, and prescription data. Data integration methods and diagnostic criteria used are described in Supplementary Data Table S2. Clinician documentation of LTCs was recorded in ICEA as free-text or coded variables. Prior to 2019, LTCs were documented exclusively as free-text. From 2019 onwards, clinicians recorded the presence or absence of 12 key LTCs – hypertension, diabetes mellitus, hepatitis B, renal disease, cancer, heart disease, venous thromboembolism, epilepsy, peripheral vascular disease, cerebrovascular disease, asthma, and mental health conditions – as standard, with other LTCs continuing to be recorded as free-text. NCD prescriptions were recorded in ICEA from 2014 onwards. Prescriptions with clear LTC indications were categorised (Supplementary Data Table S1, doi: 10.6084/m9.figshare.31431316). Medications with multiple potential indications could not be assigned. Results of serum creatinine, haemoglobin, hepatitis B surface antigen, and hepatitis C antibody were extracted from laboratory records and used to categorise chronic kidney disease (CKD), anaemia, hepatitis B, and hepatitis C, respectively. Estimated glomerular filtration rate (eGFR) was calculated from serum creatinine and CKD was defined as an eGFR <60 ml/min/1.73 m^2^ persisting for ≥90 days.^14^ Anaemia was defined as a haemoglobin <13 g/dL (men), <12 g/dL (women), or <11 g/dL (pregnant women).^15^

### LTCs and multimorbidity

The Delphi study by Ho et al. established international consensus on 59 LTCs to measure in multimorbidity research.^16^ Forty-four of these, which could be extracted from ICEA, and four additional LTCs relevant to the study context were included in our analyses (Supplementary Data Table S3, doi: 10.6084/m9.figshare.31431316). Undernutrition is identified by the WHO as a key TB co-morbidity.^7^ It is consistently associated with both increased risk of TB disease (accounting for an estimated 0.96 million TB episodes annually) and poor TB treatment outcomes.^7,17^ Given its clinical and epidemiological importance, and in keeping with the guidance of Ho et al., undernutrition was included as an LTC in our analyses. However, undernutrition and anaemia (an LTC recommended by Ho et al.) are also driven by TB disease and uncontrolled HIV. To address potential reverse causality, we conducted an analysis to separate LTCs which, at TB diagnosis, are inseparable from TB and/or HIV severity (undernutrition and anaemia; group A) and all other LTCs (group B). Multimorbidity, the primary exposure, was defined as HIV and at least one other LTC in addition to TB. Four subcategories of multimorbidity were also studied: complex multimorbidity, physical–mental multimorbidity, group B multimorbidity, and group B complex multimorbidity. Complex multimorbidity was defined as HIV and at least two other LTCs.^18^ Physical–mental multimorbidity was defined as HIV and at least one mental health condition or substance addiction.^19^ Group B multimorbidity and group B complex multimorbidity were defined as HIV and at least one or two other group B LTCs, respectively (Supplementary Data Table S4, doi: 10.6084/m9.figshare.31431316).

### Treatment outcomes

TB treatment outcomes were categorised, according to WHO 2021 guidelines, as ‘successful’, ‘unsuccessful’, or ‘not evaluated’ (Supplementary Data Table S5, doi: 10.6084/m9.figshare.31431316).^20^ Outcomes missing in ICEA were completed from paper records, where available.

### Other variables

TB drug resistance data were extracted from laboratory and clinical records. DS-TB was defined as no resistance to isoniazid or rifampicin detected on molecular or phenotypic testing (where performed) or recorded clinically, and participant treated as DS-TB. Drug-resistant TB was defined as documented resistance to rifampicin and/or isoniazid in laboratory or clinical records. Age was categorised into <35, 35–49, and ≥ 50 years. HIV viral load and CD4^+^ count from within 30 and 90 days of TB diagnosis, respectively, were used to indicate HIV control at TB diagnosis. Viral suppression was defined as an HIV viral load <200 copies/mL.^21^ CD4^+^ cell counts were categorised as <200 or ≥200 cells/µL,^21^ acknowledging limitations of measurements at TB diagnosis.

### Statistical analysis

Analyses were performed in R (v4.5.1). Estimates of the prevalence of multimorbidity and 95% confidence intervals (CIs) were calculated using the Clopper–Pearson method. Sensitivity analyses of the prevalence of LTCs and multimorbidity by alternative methods of data integration and calendar year were performed. Subgroup analyses by site of TB, age, sex, and CD4^+^ count were undertaken. The effect of multimorbidity on the risk of an ‘unsuccessful’ treatment outcome in patients with DS-TB was evaluated using a generalised linear regression model with binomial distribution, followed by marginal standardisation (g-computation) to estimate crude and adjusted risks and risk differences. An a priori directed acyclic graph identified age, sex, occupation, and smoking status as the minimal sufficient adjustment set, and distance from a health care facility as a potential effect modifier. Subgroup analysis by site of TB was performed. Sensitivity analyses using number of LTCs and group B multimorbidity as alternative exposures were conducted *post hoc*. Complete case analysis was planned if missingness among treatment outcomes was less than 5%.

### Patient and public involvement (PPI)

A PPI group of Ugandan TB survivors was consulted throughout the study, including in developing the study objectives and determining which LTCs were relevant contributors to multimorbidity in this setting.

### Ethical statement

Ethical approval was obtained from IDI Research Ethics Committee (on 23 February 2024, IDI-REC-2023-82), the University of St Andrews School of Medicine Ethics Committee (on 4 April 2024, MD17720), and the Uganda National Council of Science and Technology (on 7 May 2024, HS3888ES).

## RESULTS

A total of 993 patients were included in the cohort (Supplementary Data Figure S1, doi: 10.6084/m9.figshare.31431316). Median age was 38.3 years (interquartile range (IQR) 31.9–46.1), and 427 (43%) were female (Table 1). The majority (654, 66.1%) had pulmonary TB. TB diagnosis occurred within 30 days of HIV diagnosis in 335 patients (37.3%). Median CD4^+^ count was 163.5 cells/µL (IQR 42.0–360.5). HIV viral load was available for 204 patients, of whom 130 (63.7%) were suppressed. Ugandan HIV guidelines advise viral load monitoring to begin 6 months after antiretroviral therapy initiation.^21^ Patients with viral load data had a longer duration since HIV diagnosis than the overall cohort (median 3,393 days [IQR 1,545–4,997] vs. 144 days [IQR 7–3,158]), suggesting that this subgroup may underrepresent patients newly diagnosed with HIV. Patients excluded due to missing clinical data at TB diagnosis (31/1,024, 3%) were more recently diagnosed with HIV but otherwise comparable to the included cohort (Supplementary Data Table S6, doi: 10.6084/m9.figshare.31431316).

**Table 1.** Description of cohort, including the prevalence of long-term conditions.

Covariate	Category	n (%)
Age (years)	–	Median 38.3 (IQR 31.9–46.1)
	<35	364 (36.7)
	35–49	462 (46.5)
	≥50	167 (16.8)
	Unknown	0 (0)
Sex	Female	427 (43.0)
	Male	566 (57.0)
	Unknown	0 (0)
Occupation	Income generating activity	52 (5.2)
	Unemployed	11 (1.1)
	Unknown	930 (93.7)
Smoking status	Current smoker	14 (1.4)
	Not current smoker	385 (38.8)
	Unknown	638 (64.2)
Site of TB	Pulmonary	654 (65.9)
	Smear positive	414 (41.9)
	Smear negative	240 (24.3)
	Extra-pulmonary	335 (33.7)
	Unknown	4 (0.4)
Sputum Xpert MTB/RIF**^A^** results (pulmonary TB only, n = 654)	Positive	193 (29.5)
	Negative	55 (8.4)
	Unknown	406 (62.1)
TB drug resistance profile	DS-TB	982 (98.9)
	No drug resistance on sensitivity testing	196 (20.3)
	No sensitivity results, treated as presumed DS-TB	786 (78.5)
	DR-TB**^B^**	11 (1.1)
Duration since HIV diagnosis (days)	–	Median 144 (IQR 7–3,158)
	TB diagnosis < 30 from HIV diagnosis	335 (33.7)
	TB diagnosis ≥ 30 from HIV diagnosis	562 (56.6)
	Unknown	96 (9.7)
HIV viral load (copies/mL)	Unsuppressed (viral load ≥200)	74 (7.5)
	Suppressed (viral load <200)	130 (13.1)
	Unknown	789 (79.5)
CD4^+^ cell count (cells/µL)	–	Median: 163.5 (IQR 42.0–360.5)
	CD4^+^ count <200	454 (45.7)
	CD4^+^ count ≥200	368 (37.0)
	Unknown	171 (17.2)
Long-term conditions**^C^**	Anaemia	517 (52.1)
	Mild**^D^**	167 (16.8)
	Moderate**^D^**	260 (26.2)
	Severe**^D^**	90 (9.1)
	Missing haemoglobin result	337 (33.9)
	Undernutrition (BMI <18.5 kg/m^2^)	341 (34.3)
	Mild/moderate (BMI 16.0–18.4 kg/m^2^)	241 (24.3)
	Severe (BMI <16.0 kg/m^2^)	100 (10.1)
	Missing BMI	42 (4.2)
	Hypertension	82 (8.3)
	Alcohol use disorder	70 (7.0)
	Hepatitis B	49 (4.9)
	Obesity (BMI ≥30.0 kg/m^2^)	35 (3.5)
	Diabetes mellitus	19 (1.9)
	Chronic kidney disease	14 (1.4)
	Mental health condition	11 (1.1)
	Other**^E^**	25 (2.5)

Demographics and clinical characteristics of 993 people in the study cohort of patients with TB–HIV.

BMI = body mass index; CD4 = cluster of differentiation 4; DR-TB = drug-resistant TB; DS-TB = drug-susceptible TB; IQR = interquartile range; LTC = long-term health condition.

A Either Xpert MTB/RIF or Xpert MTB/RIF Ultra assay used.

B Rifampicin resistance on either GeneXpert or line probe assay (seven patients), rifampicin resistance clinically documented (three patients), or multidrug resistance clinically documented (two patients).

C Sensitivity analyses of the prevalence of LTCs and multimorbidity by alternative methods of data integration and calendar year are shown in Supplementary Data Figures S3 and S4, doi: 10.6084/m9.figshare.31431316.

D Anaemia graded according to the World Health Organization.^15^ Guideline on haemoglobin cut-offs to define anaemia in individuals and populations. Geneva: Mild = 11.0–12.9 g/dL for men, 11.0–11.9 g/dL for non-pregnant women, and 10.0–10.9 g/dL for pregnant women. Moderate = 8.0–10.9 g/dL for men and non-pregnant women, and 7.0–9.9 g/dL for pregnant women. Severe = <8.0 g/dL for men and non-pregnant women, and <7.0 g/dL for pregnant women.

E Recreational drug dependency (n = 6), cerebrovascular disease (n = 5), cardiovascular disease (n = 3), epilepsy (n = 3), venous thromboembolism (n = 2), physical disability (n = 2), cancer (n = 1), chronic lung disease (n = 1), chronic liver disease (n = 1), and visual impairment (n = 1).

### Prevalence and patterns of multimorbidity

The prevalence of multimorbidity was 76.1% (95% CI: 73.4%–78.8%, 756/993) (Figure 1). 34.3% (95% CI: 31.4%–37.4%, 341/993) of patients had complex multimorbidity. When restricted to group B LTCs (i.e., excluding undernutrition and anaemia), the prevalence of multimorbidity was 18.0% (95% CI: 15.7%–20.6%, 179/993) and of complex multimorbidity was 3.1% (95% CI: 2.1%–4.4%, 31/993). 8.3% (95% CI: 6.6%–10.1%, 82/993) of patients had physical-mental multimorbidity. Among the stratified results, the prevalence of multimorbidity was higher in patients with a CD4^+^ count <200 cells/µL (87.0% [95% CI: 83.6%–90.0%]) than those with CD4^+^ counts ≥200 cells/µL (70.1% [65.1%–74.7%]) – see Supplementary Data Figure S2, doi: 10.6084/m9.figshare.31431316.

**Figure 1.**
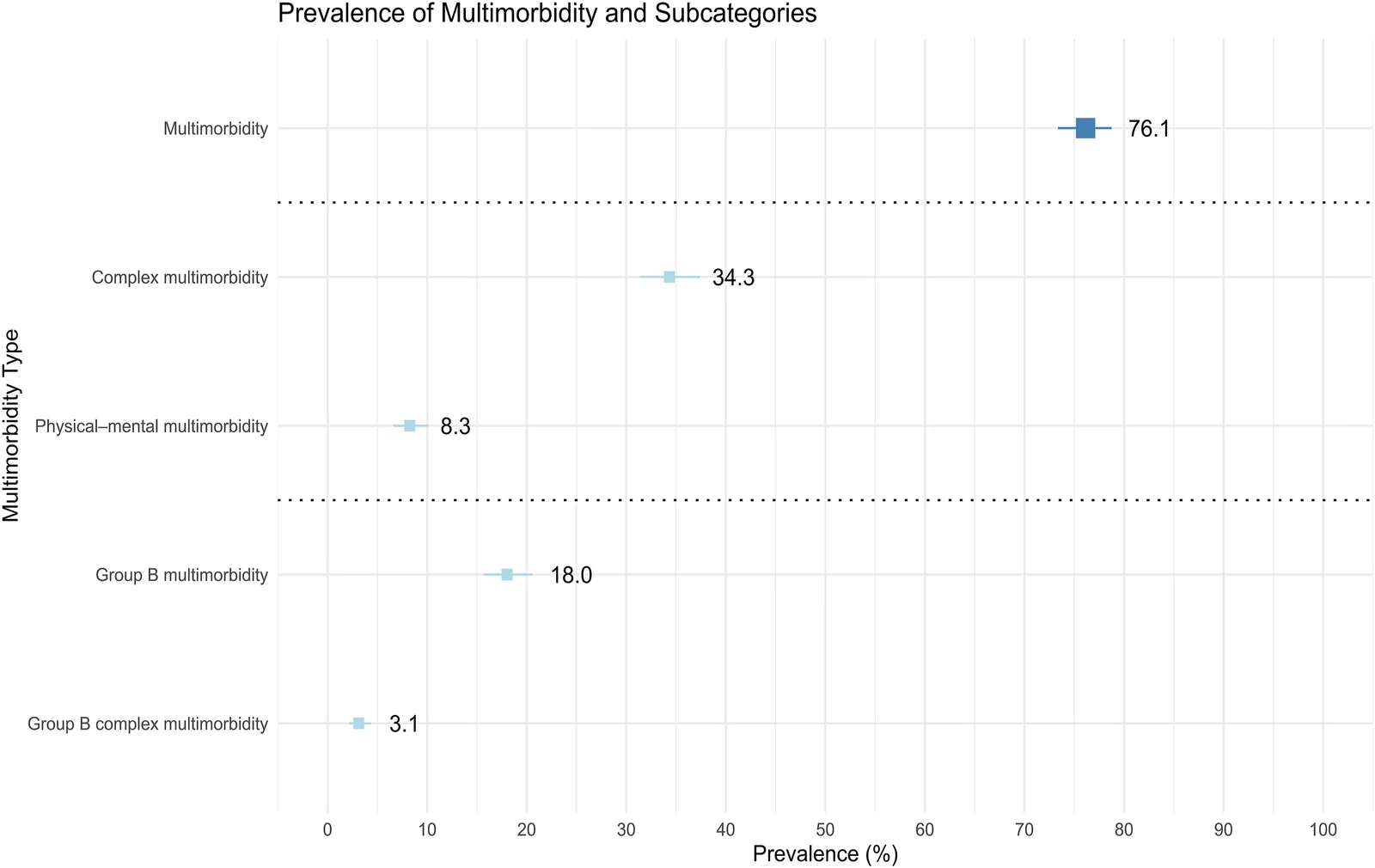
Prevalence of multimorbidity and subcategories of multimorbidity. Prevalence (boxes, numbers) and 95% confidence intervals (lines) of multimorbidity (main exposure) and subcategories of multimorbidity at the time of TB diagnosis among the cohort of patients with TB–HIV. Multimorbidity (main exposure) = HIV and at least one other long-term health condition (LTC). Complex multimorbidity = HIV and at least two other LTCs. Physical–mental multimorbidity = HIV and at least one mental health condition or substance addiction. Group B multimorbidity = HIV and at least one group B LTC. Group B complex multimorbidity = HIV and at least two other group B LTCs. Group B LTCs = LTCs excluding anaemia and undernutrition, which are inseparable from TB severity at time of TB diagnosis.

The most prevalent individual LTCs were anaemia (517/993, 52.1%), undernutrition (341/993, 34.3%), hypertension (82/993, 8.3%), and alcohol use disorder (70/993, 7.0%), see Table 1 and Figure 2. Examination of the intersection of LTCs showed that multimorbidity and complex multimorbidity largely reflected the high prevalences of anaemia and undernutrition; 63.8% (634/993) of patients with TB–HIV had anaemia and/or undernutrition, and 51.8% (514/993) without any other LTCs (Figure 2).

**Figure 2.**
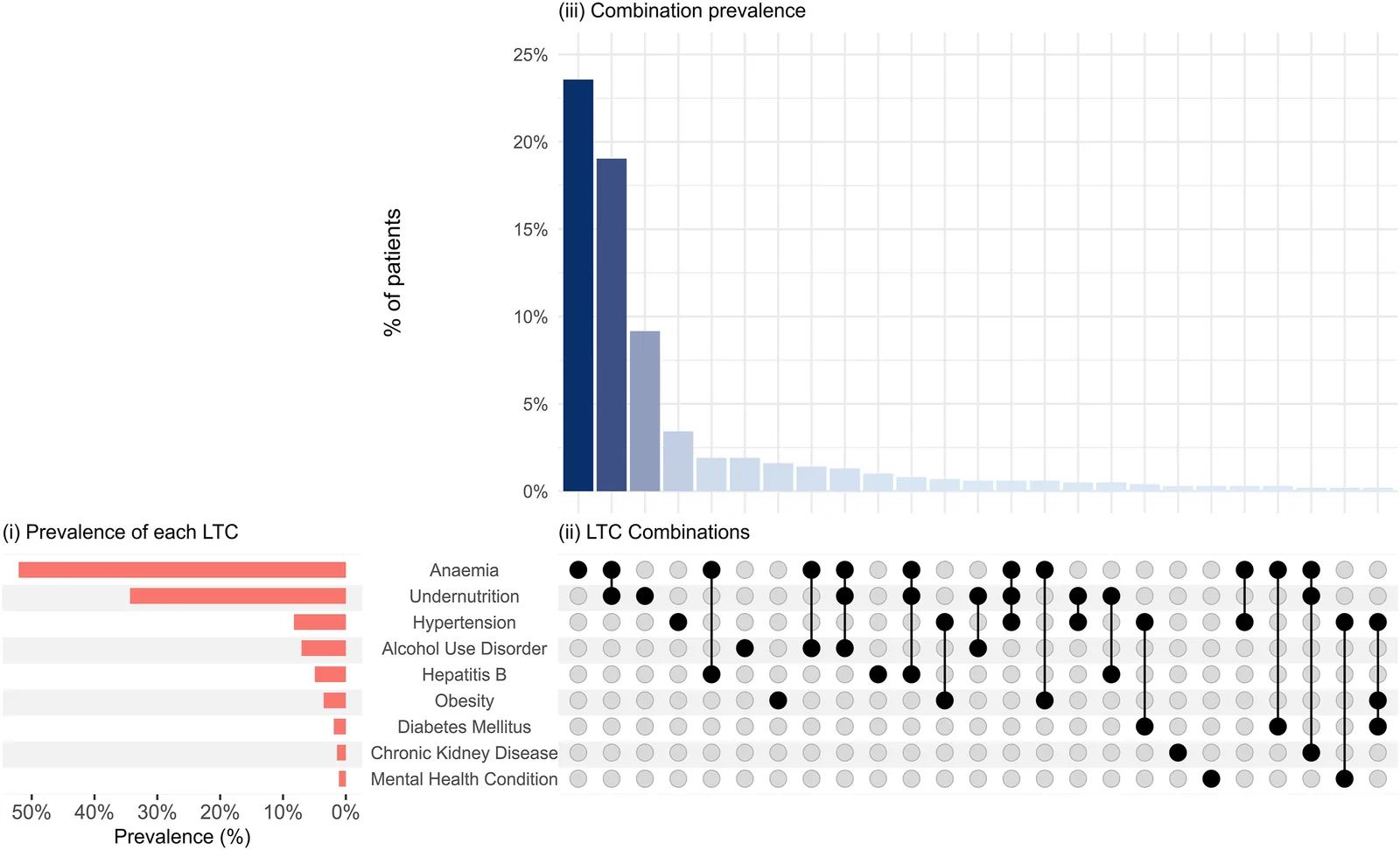
Prevalence and intersections of the most prevalent long-term conditions (LTCs). Three panels documenting the prevalence of individual and combinations of LTCs in the cohort. i) The set size plot (bottom left panel) shows the prevalence of each LTC. ii) The matrix plot (bottom right panel) shows the combinations of LTCs; filled circles indicate the conditions present in each combination, ordered by frequency. iii) The intersection plot (top right panel) shows the percentage of patients with each LTC combination represented in the matrix plot. Percentages are calculated using the full cohort as the denominator (N = 993). Only the 25 most frequent intersections are displayed. Intersections are mutually exclusive; each participant contributes to one combination only.

LTC prevalences differed by age group and CD4^+^ count. Compared with younger participants (<50 years) or those with CD4^+^ counts <200 cells/µL, older individuals and those with higher CD4^+^ counts had lower prevalences of anaemia (34.1% vs. 55.7% and 43.5% vs. 74.7%, respectively) and undernutrition (28.7% vs. 35.5% and 27.7% vs. 41.2%, respectively), but higher prevalences of several group B LTCs (see Figure 3 and Supplementary Data Table S7, doi: 10.6084/m9.figshare.31431316).

**Figure 3.**
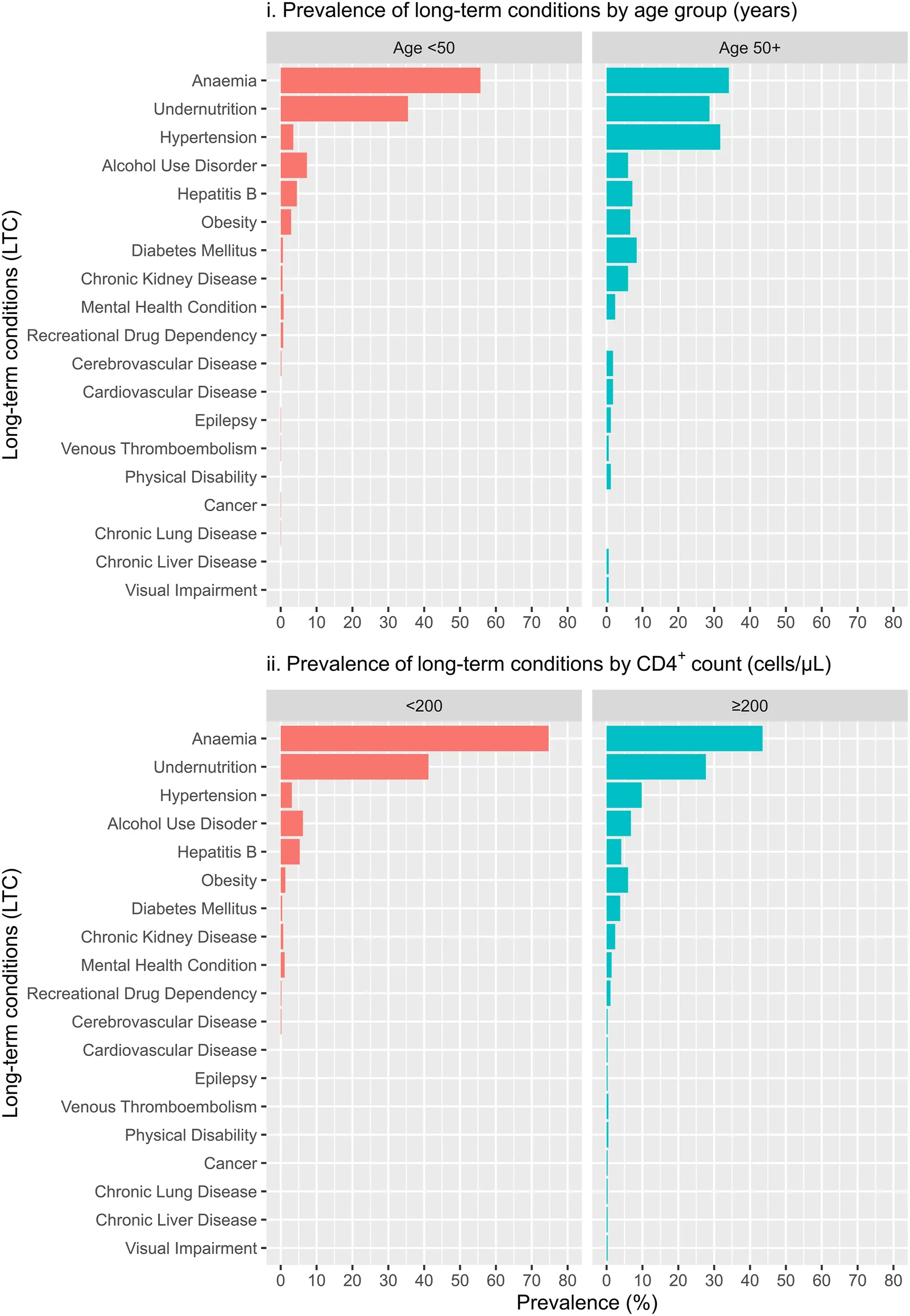
Prevalence of long-term conditions (LTC) by age group and CD4^+^ cell count. Bar charts representing the prevalence of each LTC at time of TB diagnosis; i) by age group (<50 and ≥50 years), and ii) by CD4^+^ cell count (<200 cells/µL and ≥200 cells/µL). CD4 = cluster of differentiation 4 cell count.

### Effect of multimorbidity on TB treatment outcomes in patients with DS-TB

Treatment outcome was not evaluated in only 37/982 (3.8%) patients with DS-TB (Supplementary Data Table S8, doi: 10.6084/m9.figshare.31431316); therefore, a complete case analysis was performed. Patients with multimorbidity, in its primary definition, had a 3.98% (95% CI: 0.01–7.96) higher crude risk of an unsuccessful treatment outcome. Data on distance to a health care facility (potential effect modifier) were not present in the dataset. After adjustment for age and sex only (due to substantial missingness in occupation and smoking status), patients with multimorbidity had a 3.70% higher risk of an unsuccessful treatment outcome than those without, albeit with a CI that crossed the null (95% CI: −0.35% to 7.74%, likelihood ratio test *P* = 0.09) (Table 2). The direction of effect was the same in subgroup analyses by site of TB, though these results had even wider CIs due to splitting of the dataset (Supplementary Data Table S9, doi: 10.6084/m9.figshare.31431316). There was no evidence for an association between group B multimorbidity (i.e., excluding undernutrition and anaemia) and treatment outcomes (adjusted risk difference [aRD] 1.41% [95% CI: −4.05% to 6.86%], *P* value = 0.60), see Supplementary Data Table S10, doi: 10.6084/m9.figshare.31431316. There was evidence of an association between number of LTCs and treatment outcome (*P* = 0.04); compared with HIV only, the aRD was 1.77% (−2.58% to 6.12%) for HIV plus one LTC and 6.04% (1.08%–10.99%) for HIV plus ≥2 LTCs (Supplementary Data Table S11, doi: 10.6084/m9.figshare.31431316).

**Table 2.** Association between multimorbidity and unsuccessful TB treatment outcome.

Variable	Number (n [%])	Crude			Adjusted		
		Marginal risk (%)	RD (95% CI) (%)	*P* value	Marginal risk (%)	RD (95% CI) (%)	*P* value
Multimorbidity							
No MM	236 (24.0)	6.70	Ref	*P* = 0.07	6.89	Ref	*P* = 0.09
MM	746 (76.0)	10.68	3.98 (0.01–7.96)		10.59	3.70 (−0.35–7.74)	

Marginal risks and risk differences calculated for the association between multimorbidity and unsuccessful treatment outcomes among 982 people with drug-susceptible TB. Latter model adjusted for age and sex. *P* values from likelihood ratio tests.

CI = confidence interval; MM = multimorbidity; RD = risk difference; Ref = reference category.

## DISCUSSION

Multimorbidity at TB diagnosis was common (76.1%) in this cohort of PLWHIV. Previous studies from LMICs report prevalences of multimorbidity in TB, defined as two or more LTCs additional to TB, between 1.14% and 45%.^11,12,22,23^ The comprehensive clinical, laboratory, and prescription data available within ICEA enabled inclusion of a greater breadth of LTCs in this study and has emphasised the complexity of care required at TB diagnosis, alongside initiation of anti-TB therapy (ATT).

Undernutrition is common among patients with TB in Uganda, as observed in this and prior studies.^11,24^ Providing nutritional support increases sputum conversion, serum haemoglobin, and weight gain, which is associated with reduced mortality.^25,26^ Although undernutrition still dominates in patients with TB–HIV, obesity and diabetes (observed in 3.5% and 1.9% of this cohort, respectively) are increasing in African PLWHIV.^27,28^ The findings from this cohort reinforce the importance of integrating nutritional support into TB care, while recognising that individualised management will increasingly be required to address the diversity of nutritionally driven conditions in TB–HIV populations. At TB diagnosis, cross-sectional data cannot differentiate anaemia and undernutrition as markers of TB severity versus independent conditions with distinct pathophysiological or socio-economic drivers. Defining them instead by their duration would have introduced follow-up bias. To reflect the health care needs of patients at TB diagnosis, we included both in their cross-sectional definitions. Both were highly prevalent at TB diagnosis and have been shown in other studies to often require ongoing management after TB treatment completion.^29,30^ Even with anaemia and undernutrition excluded, one in five patients with TB–HIV in this cohort had at least one other LTC.

The pattern of multimorbidity differed in older patients in this cohort with a shift away from undernutrition and anaemia and towards NCDs associated with ageing and epidemiological transition, such as hypertension and diabetes. This emphasises that a ‘one-size-fits-all’ approach to national TB programmes may not serve older patients well.^31^

Globally, 88% of patients with DS-TB achieve treatment success.^6^ WHO TB treatment outcomes, which focus on ATT completion, with or without evidence of mycobacterial death, do not capture complexity in recovery trajectories nor post-TB sequelae. Nevertheless, there was a direction of effect towards increased risk of an unsuccessful TB treatment outcome with multimorbidity, though the estimate was small and imprecise. An effect of number of LTCs on risk of unsuccessful outcome was seen, albeit with wide CIs for some strata. Future studies exploring the effect of multimorbidity on TB treatment outcomes should also measure, as standard, patient-centred and post-treatment outcomes.

A key strength of this study is the longitudinal, multifaceted ICEA dataset which enabled LTC classification across clinical, laboratory, and prescription data. This mitigates against underreporting frequently seen in studies using routinely collected data. The wide range of LTCs captured provides a stronger estimate of multimorbidity compared to studies restricted to fewer LTCs. Nevertheless, the prevalence of multimorbidity is likely underestimated. Some key conditions, including chronic lung and cardiovascular disease, relied solely on clinical documentation, and mental health conditions were less prevalent than in prospective Ugandan data (1.1% vs. 23.7%).^32^ Underestimation of the prevalence of multimorbidity would be expected to attenuate, and increase uncertainty around, its effect on treatment outcome. Missing data prevented adjustment by occupation and smoking status (2/4 of the confounders from our minimal sufficient adjustment set) and inclusion of distance from health care facility as a potential effect modifier. CD4^+^ count was used to define advanced HIV disease, but values at TB diagnosis may be influenced by intercurrent illness and may not reflect HIV stage. As this study was conducted in a specialist urban referral centre, findings may not be generalisable to other clinics in Kampala or rural settings.

## CONCLUSION

There was a high prevalence of multimorbidity in PLWHIV at time of TB diagnosis in this study in Kampala, Uganda. In patients with multimorbidity, there may be an increased risk of an unsuccessful TB treatment outcome, but the CI crossed the null. Our results highlight the need for holistic care for patients with TB–HIV.
